# Anti-Zika virus and anti-Usutu virus activity of human milk and its components

**DOI:** 10.1371/journal.pntd.0008713

**Published:** 2020-10-07

**Authors:** Rachele Francese, Andrea Civra, Manuela Donalisio, Nicola Volpi, Federica Capitani, Stefano Sottemano, Paola Tonetto, Alessandra Coscia, Giulia Maiocco, Guido E. Moro, Enrico Bertino, David Lembo

**Affiliations:** 1 Department of Clinical and Biological Sciences, Laboratory of Molecular Virology and Antiviral Research, University of Turin, Orbassano (TO), Italy; 2 Department of Life Sciences, University of Modena and Reggio Emilia, Modena, Italy; 3 Department of Public Health and Pediatrics, Neonatal Intensive Care Unit, University of Turin, Turin, Italy; 4 Italian Association of Human Milk Banks (AIBLUD), Milan, Italy; Baylor College of Medicine, UNITED STATES

## Abstract

The benefits of human milk are mediated by multiple nutritional, trophic, and immunological components, able to promote infant’s growth, maturation of its immature gut, and to confer protection against infections. Despite these widely recognized properties, breast-feeding represents an important mother-to-child transmission route of some viral infections. Different studies show that some flaviviruses can occasionally be detected in breast milk, but their transmission to the newborn is still controversial. The aim of this study is to investigate the antiviral activity of human milk (HM) in its different stages of maturation against two emerging flaviviruses, namely Zika virus (ZIKV) and Usutu virus (USUV) and to verify whether HM-derived extracellular vesicles (EVs) and glycosaminoglycans (GAGs) contribute to the milk protective effect.

Colostrum, transitional and mature milk samples were collected from 39 healthy donors. The aqueous fractions were tested *in vitro* with specific antiviral assays and EVs and GAGs were derived and characterized. HM showed antiviral activity against ZIKV and USUV at all the stages of lactation with no significant differences in the activity of colostrum, transitional or mature milk. Mechanism of action studies demonstrated that colostrum does not inactivate viral particles, but it hampers the binding of both flaviviruses to cells. We also demonstrated that HM-EVs and HM-GAGs contribute, at least in part, to the anti-ZIKV and anti-USUV action of HM.

This study discloses the intrinsic antiviral activity of HM against ZIKV and USUV and demonstrates the contribution of two bioactive components in mediating its protective effect. Since the potential infectivity of HM during ZIKV and USUV infection is still unclear, these data support the World Health Organization recommendations about breast-feeding during ZIKV infection and could contribute to producing new guidelines for a possible USUV epidemic.

## Author summary

ZIKV and USUV are emerging flaviviruses that cause conditions ranging from mild febrile diseases to more sever outcomes. ZIKV is associated with microcephaly in newborns and USUV neurotropism represents a growing concern for human health. We studied these viruses in the context of breast-feeding. Breast-milk is a complex biofluid to nourish infants, support their growth and to protect them from numerous diseases, but it also represents a transmission route of several infections. It has been reported that flaviviruses can occasionally be detected in breast-milk, with limited information existing about their possible transmission through breast-feeding. We therefore explored the intrinsic protective role of human milk against ZIKV and USUV infections *in vitro* and we also assessed the contribution of specific components in mediating this activity. We demonstrated that human milk is endowed with anti-ZIKV and anti-USUV activity at all maturation stages and that it acts by altering virus attachment to the host cell. This activity is mostly due to non-specific bioactive factors, including extracellular vesicles and glycosaminoglycans. Our findings support the use of fresh milk (or from donor banks) as the food of choice for nutrition and protection of newborns in a possible context of ZIKV or USUV epidemics.

## Introduction

Zika virus (ZIKV) and Usutu virus (USUV) are two emerging flaviviruses mostly transmitted by mosquitos. *Aedes aegypti* and *Culex pipiens* mosquitos are their major vectors respectively [1–3]. Most ZIKV and USUV infections are asymptomatic, but in symptomatic cases, they may cause conditions ranging from a mild febrile disease to more severe outcomes with neurological involvement.

ZIKV infection has been associated with the Guillain-Barré syndrome in adults and with a variety of neurological impairments, including microcephaly, in infants born to infected mothers [4,5]. It has caused a series of epidemics in the Americas, Asia and the Pacific in the past decade and it is now considered an important public health concern [6]. To date, 84 countries and territories have reported autochthonous transmission of ZIKV [7].

The less-known USUV has recently attracted the attention of the scientific community due its potential for emergence and its extensive spread in Europe [8,9]. It is phylogenetically closely related to West Nile virus (WNV) and it is maintained through an enzootic cycle between migratory birds and ornithophilic mosquitos, with humans representing incidental hosts [9]. Seroprevalence studies suggested that USUV infections in humans may have been largely underestimated, and many of them may be asymptomatic. The full clinical presentation of human severe USUV infection is still partially unknown, but cases of meningoencephalitis and facial paralysis have been reported and its neurotropism represents a growing concern for human health [10–12].

No antivirals or vaccines are currently available against either virus and the only way to prevent these infections is to avoid mosquito bites.

We studied ZIKV and USUV in the context of breastfeeding. Previous reports have shown that some flaviviruses, such as Dengue virus (DENV), WNV and Yellow fever virus (YFV), can occasionally be present in breast milk [13]. In particular, ZIKV RNA has been reported in breast milk from 3 to 33 days after maternal onset of fever and ZIKV infectious particles were also detected in this biofluid [14–18]. Nevertheless, this mode of transmission to the newborn is still controversial [19–25]. Probably due to the small number of human cases, the presence of USUV in breast milk is currently unknown, but its strong correlation with WNV suggests it could be possible. Notwithstanding these evidences, the short and long-term health advantages of breastfeeding for both neonate and lactating mother outweigh any potential risk of transmission [26]. The World Health Organization (WHO) recommends indeed that mothers with possible or confirmed ZIKV infection continue to breastfeed [27].

Breast milk composition is extremely complex, individual-specific and variable according to the stage of lactation. It includes macro- and micronutrients and a wide variety of non-nutritional bioactive components [28]. Amongst the latter, secretory IgA (sIgA), toll-like receptors (TLRs), lactoferrin, lactadherin, oligosaccharides (HMOs) support the development of the immature immune system of the neonate and confer intrinsic protection against infections [29]. Therefore, HM is a possible source of viral infections, but these substances could directly affect viral infectivity. In the case of flaviviruses, DENV and Japanese encephalitis virus (JEV) are neutralized by the lipid fraction of breast milk and ZIKV and hepatitis C virus (HCV) are destroyed by free-fatty acids released upon storage by milk lipases in a time-dependent manner [30–33]. Herein, we aimed to explore the intrinsic anti-ZIKV and anti-USUV activity of human milk, according to its maturation stage and regardless of the storage affected-lipid fraction.

We also investigated the antiviral contribution of human milk-derived extracellular vesicles (HM-EVs) and human-milk glycosaminoglycans (HM-GAGs). Briefly, EVs are lipid enclosed vesicles, ranging from 30 to 1000 nm in diameter, that are released by most tissues including breast epithelial cells, macrophages and lymphocytes present in breast milk [34–36]. These vesicles can selectively be taken up by other cells, in which they release their molecular cargo (e.g. DNA, RNAs, enzymes, signalling proteins), playing a role in intercellular signalling, immune response, stem cell differentiation, tissue regeneration and viral replication [37,38]. GAGs are other abundant constituents of human milk defined as linear heteropolysaccharides composed of repeating disaccharidic units [39–41]. Detailed analyses performed on HM-GAGs demonstrated the presence of a complex mixture made up of chondroitin sulfate (CS)/dermatan sulfate (DS), heparan sulfate (HS)/heparin (Hep) and a minor percentage of hyaluronic acid (HA), with the CS/DS fraction being the most represented (⁓ 55%) followed by HS/Hep (⁓ 40%) [39].

HM-GAGs and HM-EVs have recently become the subject of increasing interest for their implication for infants [42,43]. HM-EVs have been demonstrated to be active *in vitro* against human immunodeficiency virus (HIV) [44] and HM-GAGs have also shown anti-bacterial and anti-viral activity due to their ability to act as soluble receptors inhibiting the attachment of different pathogens to the intestinal mucosa [45–47]. Both human milk constituents, have been poorly investigated for their antiviral action so far, therefore their role needs to be clarified.

Here we report that human milk is endowed with anti-ZIKV and anti-USUV activity at all maturation stages and that it acts by altering virus attachment to the host cell. This activity is mostly due to non-specific bioactive factors, including HM-EVs and HM-GAGs.

## Methods

### Ethic statement

An ethical review process was not required for this study since it was not a clinical trial. Each milk donor involved in this research signed a written consent form, where the mother's and infant's data protection was assured. Moreover, the donors were informed about the study design.

### Human milk sample collection and clarification

Thirty-nine healthy mothers were enrolled in the study: 18 mothers donated as many colostrum samples (days 1–5 postpartum), 11 mothers donated colostrum, transitional (days 6–14 postpartum) and mature milk samples (beyond day 15 postpartum), and 10 mothers each donated 15 ml of mature milk that were added in a unique pool. All mothers were admitted to Sant’Anna Hospital (Città della Salute e della Scienza of Turin, Italy). The donors cleaned their hands and breasts according to the Italian HMB guidelines [48], and the milk samples were collected in sterile bisphenol-free polypropylene bottles using a breast pump and immediately stored at -20°C unless otherwise stated. After thawing, the milk samples were centrifuged at a low speed (2000 x *g*) for 10 minutes at room temperature to remove the fat globule layer. The defatted milk was then transferred to a new tube and centrifuged at 12000 x *g* for 30 minutes to obtain the aqueous fraction. The supernatant was filtered through a syringe, equipped with a 0.45 *μ*m pore size sterile filter (Sarstedt, Verona, Italy), to further eliminate any cells and cellular debris.

### Cell lines

African green monkey fibroblastoid kidney cells (Vero) (ATCC CCL-81) were cultured in Eagle’s minimal essential medium (MEM; Sigma, St. Louis, MO) supplemented with heat-inactivated, 10% (v/v) fetal bovine serum (FBS) (Sigma). The embryonic human kidney cells (293T) (ATCC CRL-3216) were grown as monolayer in Dulbecco's modified Eagle's medium (DMEM; Sigma) supplemented with 10% FBS and 1% Glutamax-I (Invitrogen, Carlsbad, CA) and low-passage-number (<30) human foreskin fibroblasts (HFF-1) (ATCC SCRC-1041) were grown as monolayers in DMEM supplemented with 15% FBS. The media were supplemented with 1% (v/v) antibiotic-antimycotic solution (Zell Shield, Minerva Biolabs, Berlin, Germany) and cells were grown at 37°C in an atmosphere of 5% of CO2.

The antiviral assays against ZIKV and USUV were performed on Vero cells using MEM supplemented with 2% of FBS, unless otherwise stated.

### Viruses

Two strains of infectious Zika viruses (1947 Uganda MR766 and 2013 French Polynesia HPF2013) were generated by transfection of 293T cells with two plasmids (pCDNA6.2 Zika MR766 Intron3115 HDVr MEG 070916 5 and pCDNA6.2 Zika HPF2013 3864,9388Intron HDVr MEG091316 2) as previously described [49]. The viruses were then propagated in Vero cells and titrated by plaque assay. All the antiviral assays were performed with ZIKV HPF2013 strain, unless otherwise stated.

Usutu virus (Strain: 3345 Isolate: Arb276) was isolated and produced by APHA (Animal & Plant Health Agency–GOV. UK) and kindly provided by the European Viral Archive Global (EVAg). It was propagated in Vero cells and titrated by means of the indirect immunoperoxidase staining procedure, by using a mouse monoclonal antibody direct to flavivirus protein E (D1-4G2-4-15 (4G2), Novus Biological) and a secondary antibody peroxidase-conjugated AffiniPure F(ab’)2 Fragment Goat Anti-Mouse IgG (H+L) (Jackson ImmunoResearch Laboratories Inc., 872 W. Baltimore Pike, West Grove, PA 19390).

### Extracellular vesicles (EVs) isolation and characterization

Extracellular vesicles were extracted from the aqueous fraction of three colostrum samples before their freezing. The aqueous fraction was obtained as described above and subsequently incubated with 2:1 v/v ratio of colostrum:ExoQuick solution (System Biosciences, CA) overnight at 4°C. The extracellular vesicles were then purified, according to the manufacturer’s instructions. The EV pellet was resuspended in 1 x PBS, quantified to establish the protein concentration using a protein assay kit (Bio-Rad Laboratories, Munich), aliquoted and stored at -80°C until use.

The EV protein profile was analyzed by means of western blotting. HFF cell lysate was used to verify the reactivity of the anti-calnexin primary antibody. These cells were chosen as control since they are a reliable and highly standardized human cell line. A RIPA buffer, containing protease inhibitors, was added to the EV pellet, or to the HFF cells, for 10 minutes at RT to allow complete lysis. Soluble proteins were collected, by means of centrifugation at 15,000 *x g*, and were then quantified using a protein assay kit. The western blot was performed as previously described [50]. Primary antibodies: anti-CD63, anti-CD9, anti-CD81, anti-calnexin, anti-Hsp70 and anti-caveolin1; secondary antibodies: anti-rabbit and anti-mouse (System Biosciences).

A nanoparticle tracking analysis system (NTA) (NanoSight NS300, Malvern Instruments Ltd., UK) was used to determine particle size and particle concentration per milliliter at the ideal particle per frame value (63–65 particles/ frame).

### Human milk glycosaminoglycans (HM-GAGs) isolation and characterization

50 mL of mature milk were defatted with acetone. After centrifugation at 10,000 g for 15 min and drying at 60°C for 24 h, the pellet was solubilized in 200 mL of distilled water and treated with 100 mg of pancreatin (Sigma-Aldrich, code 1071301000, 350 FIP-U/g Protease, 6000 FIP-U/g Lipase, 7500 FIP-U/g Amylase) at 60°C for 24 h in a stirrer. After boiling for 10 min and centrifugation at 5,000 g for 20 min, three volumes of ethanol were added to the supernatant and the mixture stored at 4°C for 24 h. After centrifugation at 10,000 g for 15 min and dried at 60°C for 6 h, the dried powder was dissolved in 100 ml of 50 mM NaCl and centrifuged at 10,000 g for 10 min. The supernatant was applied to a column (5 x 10 cm) packed with QAE Sephadex A-25 anion-exchange resin equilibrated with the same NaCl solution. GAGs were eluted with a linear gradient of NaCl from 50 mM to 2.0 M from 0 to 200 min using low-pressure liquid chromatography (Biologic LP chromatography system from BioRad) at a flow of 1 ml/min. Fractions positive to uronic acid assay were collected [51]. Three volumes of ethanol were added to the pooled fractions and stored at 4°C for 24 h. The precipitate was centrifuged and dried at 60°C. The dried purified HM-GAGs were dissolved in distilled water and lyophilized for the virus inhibition assay or further analysis.

For the antiviral assays, GAGs were dissolved in PBS and stocked at 4°C until use.

HM-GAGs composition was evaluated by electrophoresis on acetate of cellulose [52]. The purity of the milk extract was evaluated by measuring the protein content by the Folin-Ciocalteu test (Sigma-Aldrich, code MAK365-1KT) and the GAGs content by the uronic acid assay [51].

Structural characterization of the CS/DS and HS/Hep components of HM-GAGs was performed by determining the corresponding constituent disaccharides. Briefly, HM-GAGs were treated with chondroitinase ABC or chondroitinase AC for 10 h at 37°C in 50 mM Tris-Cl pH 8.0 to produce the CS/DS constituent disaccharides. HM-GAGs were also incubated with a cocktail of heparinases (heparinases I, II and III) in 0.1 M sodium acetate/calcium acetate pH 7.0 at 38°C overnight to release the HS/Hep disaccharides. The unsaturated disaccharides produced were derivatized with 2-Aminoacridinone (AMAC) as previously described [39] and the fluorotagged disaccharides separated and analysed by capillary electrophoresis equipped with a Laser-Induced Fluorescence (LIF) detector [53]. By this analytical approach we also determined the HA content besides the structural composition and charge density of the sulphated heteropolysaccharides CS/DS and HS/Hep.

### Virus inhibition assay

The anti-ZIKV and anti-USUV activity of human milk (colostrum, transitional or mature milk) was determined by means of plaque reduction assay or focus reduction assay respectively.

Vero cells were seeded at a density of 6,5x10^4^ cells /well in 24 well plate for ZIKV antiviral assays or at a density of 1,3x10^4^/well in 96 well plate for USUV antiviral assays. The following day, cells were pre-treated with serial dilutions of human milk aqueous fraction (from 1:3 to 1:6561 parts) for 1h at 37°C. The virus was pre-treated under the same experimental conditions simultaneously: mixtures of serial dilutions of human milk and the same amount of virus were incubated for 1 h at 37°C at multiplicities of infection (MOIs) of 0.0005 PFU/cell for ZIKV and 0.02 FFU/cell for USUV. After a gentle wash, these mixtures were added to cells for 2h at 37°C. Subsequently, the ZIKV infected cells were washed twice with warm medium and overlaid with a 1.2% methylcellulose medium for 72 h at 37°C. In the case of USUV, infected cells were washed twice and overlaid with fresh medium for 24h at 37°C.

The number of ZIKV plaques were counted after cell fixation and staining with a solution of 0.1% crystal violet in 20% ethanol. The USUV-infected cells were detected by means of indirect immunostaining as described above. The inhibitive dilution that produced a 50% reduction of ZIKV or USUV infection (ID_50_) was determined by comparing the treated with the untreated wells. GraphPAD Prism 8.0 software (San Diego, CA) was used to fit a variable slope-sigmoidal dose-response curve and calculate the ID_50_ values.

### Viability assay

Cell viability was measured using the MTS [3-(4,5-dimethylthia-zol-2-yl)-5-(3-carboxymethoxyphenyl)-2-(4-sulfophenyl)-2H-tetra-zolium] assay. Confluent Vero cells were treated with serial dilutions of colostrum or HM-EVs or HM-GAGs under the same experimental conditions of the virus inhibition assay. Cell viability was determined using the Cell Titer 96 Proliferation Assay Kit (Promega, Madison, WI, USA) according to the manufacturer's instructions. Absorbances were measured using a Microplate Reader (Model680, BIORAD) at 490 nm. The effect on cell viability at different dilutions of colostrum was expressed as a percentage, by comparing absorbances of treated cells with those of cells incubated with culture medium alone. The 50% cytotoxic concentrations (CC_50_) was determined using Prism software.

### Immunofluorescence assay

Subconfluent Vero cells plated on coverslips in 24-well plates were treated with colostrum (ID_90_) following the virus inhibition assay protocol. The infection was performed with MOI of 3 for both viruses. After 30 h or 24 h for ZIKV or USUV infected cells respectively, cells were washed twice with PBS and then fixed in 4% PAF for 15 min RT. Cells were permeabilized in PBS with Triton 0,1% for 20 minutes on ice and then blocked with 5% BSA for 30 minutes. Next the incubation with the primary antibody (Anti-dsRNA mAb, SCICONS J5 or anti-flavivirus protein E mAb D1-4G2-4-15 (4G2), Novus Biological) diluted in blocking buffer was performed for 1 h RT. After three washes in PBS with 0.05% Tween 20, the secondary antibody (Goat Anti-Mouse IgG Rhodamine conjugated, Santa Cruz Biotechnology) diluted in blocking buffer was added to cells for 1 h RT. Subsequently, three washes with PBS were performed and coverslips were mounted and analyzed on a confocal fluorescence microscope (LSM510, Carl Zeiss, Jena, Germany).

### Virus inactivation assay

Approximately 10^6^ FFU of ZIKV or USUV were incubated with 100 μl of colostrum for 2h at 37°C. As control, the same number of viral particles was incubated with fresh medium. After the incubation, both treated and untreated viruses were titrated to the non-inhibitory dilution of colostrum. The residual viral infectivity was determined by plaque assay (ZIKV) or by indirect immunostaining (USUV). Statistical analysis was performed using Student’s t-test. Significance was reported for p-values <0.05.

### Pre-treatment assay

Confluent Vero cell monolayers in 24 well plate (for ZIKV test) or in 96 well plate (for USUV test) were pre-treated with serial dilutions of colostrum (from 1:3 to 1:6561) for 2 h at 37°C. After washing, cells were infected with ZIKV (MOI = 0.0005) or USUV (MOI = 0.02) for 2h 37°C. The viral inoculum was then removed and two gentle washes were performed. The ZIKV infected cells were overlaid with 1.2% methylcellulose medium for 72 h at 37°C and the USUV infected cells were incubated with fresh medium for 24 h at 37°C. At the end of the incubation cells were fixed and stained with 0.1% crystal violet in 20% ethanol to count the number of ZIKV plaques or fixed and stained with indirect immunostaining to evaluate the number of USUV infected cells.

Where possible, the ID_50_ values were calculated by means of a regression analysis, using dose–response curves generated by GraphPad Prism version 8.0.

### Binding assay

Vero cells were seeded in 24 well plate at a density of 1.1x10^5^ cells/well. The following day, cells and viruses (ZIKV or USUV, MOI = 3) were cooled to 4°C for 10 minutes. The viruses were then allowed to attach to the cells in the presence of colostrum (ID_90_). After an incubation of 2 h on ice, the cells were washed with a cold medium to remove any unbound virus. The cells were then subjected to three rounds of freeze-thawing to release any bound virus, and the lysate was clarified by means of low speed centrifugation for 10 minutes. The cell-bound virus titers were determined by means of plaque assay (ZIKV) or indirect immunostaining (USUV), as outlined above. The presence of any significant differences was assessed by means of Student’s t-test, using PRISM 8.0 GraphPad Software.

### Entry assay

The Vero cells were cultured to confluence in 24-well or 96-well trays. ZIKV (MOI = 0.005) and USUV (MOI = 0.2), which had been cooled to 4°C, were allowed to attach to pre-chilled cells on ice for 2 h at 4°C. Unbound viruses were then washed, serial dilutions of colostrum (from 1:3 to 1: 6561) were added to cells and the plates were incubated at 37 °C to allow virus entry. After the viral entry, the treatment was aspirated and viral particles still present on the cell surface were inactivated by a wash with citrate buffer (citric acid 40 mM, potassium chloride 10 mM, sodium chloride 135 mM, pH 3) for 1 minute at room temperature, as previously described [49]. Cells were then washed with warm medium 3 times and overlaid with 1.2% methycellulose medium (ZIKV entry assay) for 72h or with fresh medium (USUV entry assay) for 24h. Cells were fixed and stained with 0.1% crystal violet in 20% ethanol to count the number of ZIKV plaques or fixed and stained with the indirect immunostaining procedure to evaluate the number of USUV infected cells. The viral entry blockade was determined and expressed as the mean percentage of the untreated control ± SEM. Where possible, the ID_50_ values were calculated by means of regression analysis using dose–response curves (GraphPad Prism 8.0)

### Post-entry assay

Vero cells were seeded in 96-well plates at a density of 1.3x10^4^ cells/well. The following day, the viral inoculum (ZIKV at MOI = 0.005 or USUV at MOI = 0.2) was added to cells for 2 h at 37°C. The unpenetrated viruses were inactivated with citrate buffer for 1 minute at room temperature and cells were then washed with warm medium 3 times and incubated with serial dilutions of colostrum (from 1:3 to 1:6561) for 3 h at 37°C. Finally, after 2 gentle washes, Vero cells were incubated with warm MEM for 30 h (ZIKV) or 24 h (USUV) at 37°C. The number of infected cells was determined by indirect immunostaining and the viral inhibition expressed as the mean percentage of the untreated control ± SEM. Where possible, the ID_50_ values were calculated by means of regression analysis using dose–response curves (GraphPad Prism 8.0)

### Evaluation of HM-EVs and HM-GAGs antiviral activity: virus inhibition assay

The antiviral activity of colostrum-derived EVs and of HM-GAGs was evaluated by means of the same experimental protocol described under the “virus inhibition assay” subheading. The anti-ZIKV and anti-USUV activity of HM-EVs and HM-GAGs was determined by means of plaque reduction assay (ZIKV) or focus reduction assay (USUV). Vero cells were seeded at a density of 6,5x10^4^ cells /well in 24 well plate for ZIKV antiviral assays or at a density of 1,3x10^4^/well in 96 well plate for USUV antiviral assays. The following day, cells were pre-treated with serial dilutions of HM-EVs (from 423 μg/ml to 0.19 μg/ml) or with dilutions of HM-GAGs (from 10 mg/ml to 0.1 mg/ml) for 1 h at 37°C. The viruses were pre-treated under the same experimental conditions simultaneously: mixtures of serial dilutions HM-EVs or HM-GAGs and the same amount of virus (MOI of 0.0005 PFU/cell for ZIKV and 0.02 FFU/cell for USUV) were incubated for 1 h at 37°C. After a gentle wash, these mixtures were added to cells for 2h at 37°C. Subsequently, the ZIKV infected cells were washed twice with warm medium and overlaid with a 1.2% methylcellulose medium for 72 h at 37°C. In the case of USUV, infected cells were washed twice and overlaid with fresh medium for 24h at 37°C. The number of ZIKV plaques were counted after cell fixation and staining with a solution of 0.1% crystal violet in 20% ethanol. The USUV-infected cells were detected by means of indirect immunostaining. The effective concentration that produced a 50% reduction of ZIKV or USUV infection (EC_50_) was determined by comparing the treated with the untreated wells. GraphPAD Prism 8.0 software was used to fit a variable slope-sigmoidal dose-response curve and calculate the EC_50_ values.

### Data analysis

All the results are presented as the mean values of two independent experiments. The ID_50_ values of the inhibition curves were calculated from a regression analysis using GraphPad Prism software, version 8.0 (GraphPad Software, San Diego, California, the U.S.A.) by fitting a variable slope-sigmoidal dose-response curve. Statistical analysis was performed using Student’s t-test, ANOVA Analysis of variance or the F-test, as reported in the Figure legends.

## Results

### Human milk is endowed with an intrinsic anti-ZIKV and anti-USUV activity

The first set of experiments was performed in order to investigate the intrinsic antiviral activity of human colostrum against ZIKV and USUV. Colostrum samples were collected from healthy donor mothers admitted to Sant’Anna Hospital of Turin for term or preterm delivery (Table 1).

**Table 1:** Main clinical characteristics of the first study group.

Sample n°	Gestational Age	Mother’s Age	Parity	Type of delivery
1	35+0	39	2002	CS
2	32+1	33	0000	CS
3	29+1	32	0000	CS
4	39+4	33	1001	CS
5	29+5	38	0000	S
6	38+5	40	0000	CS
7	33+2	35	0000	CS
8	27+3	43	1001	CS
9	30+0	30	1001	CS
10	37+4	35	0000	CS
11	38+4	39	1021	S
12	38+2	39	1001	S
13	38+4	37	0000	S
14	34+1	30	1001	S
15	37+4	23	0000	S
16	38+2	42	0000	CS
F1	40+1	32	0000	S
F2	33+3	39	1001	S

S: spontaneous delivery; CS: cesarean section; F1, F2: Fresh colostrum samples.

**Fig 1:**
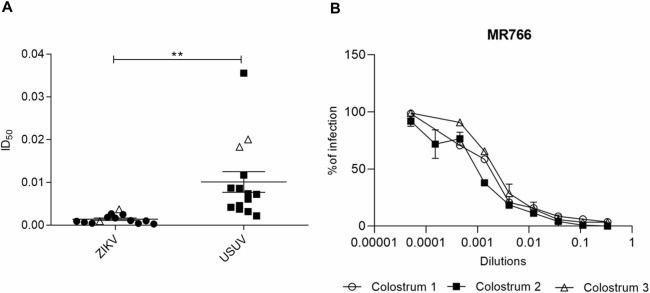
Anti-ZIKV and anti-USUV activities of defatted colostrum samples. Cells and viruses were treated before and during the infection with serial dilutions of human colostrum aqueous fraction (from 1:3 to 1:6561 parts). (A) Anti-ZIKV and anti-USUV inhibitory dilution-50 values obtained from three independent experiments are reported. Results are expressed as mean ± SEM of inhibitory dilution-50 values (Student’s t test; ** p<0.01). Results obtained with fresh colostra are indicated with white triangles. (B) Panel B shows the anti-ZIKV activity of three colostrum samples against the African MR766 strain. The dose-response curves are reported. Data are presented as % of control. Values are means ± SEM of three independent experiments performed in duplicate.

The aqueous fraction of these samples was selected as preferred biological matrix, due to its previously described lower impact on cell viability [54], and tested *in vitro* against ZIKV (HPF2013) and USUV. Briefly, cells and viruses were treated with serial dilutions of human colostrum before and during the infection. As reported in Fig 1A and S1 Table, all colostrum samples exhibited antiviral activity against both viruses although to a different extent from mother to mother. The ID_50s_ were ranging from 0.0003 to 0.0026 for ZIKV and from 0.0022 to 0.0355 for USUV indicating that human colostrum is significantly more active against ZIKV. The ID_50s_ obtained with fresh colostra, i.e. with colostra that were clarified and tested *in vitro* within 1 hour after collection, are comparable to those obtained from frozen samples (S1 Fig). Since an incomplete gestational period can affect the maturity of the mammary gland and its ability to secrete milk with the proper composition for the newborn's condition, we stratified the results comparing the antiviral activity of human colostrum from term and preterm mothers, but no significant difference was observed for both viruses (S2 Fig). Furthermore, the antiviral activity of human colostrum against different ZIKV strains was verified by testing 3 colostrum samples against ZIKV MR766 belonging to the African lineage. As shown in Fig 1B, the human colostrum inhibits the MR766 infectivity too, with ID_50_s comparable to those obtained with the microcephalic HPF2013 strain (ID_50 Colostrum1_ = 0.0012; ID_50 Colostrum2_ = 0.0009; ID_50 Colostrum3_ = 0.0023).

**Fig 2:**
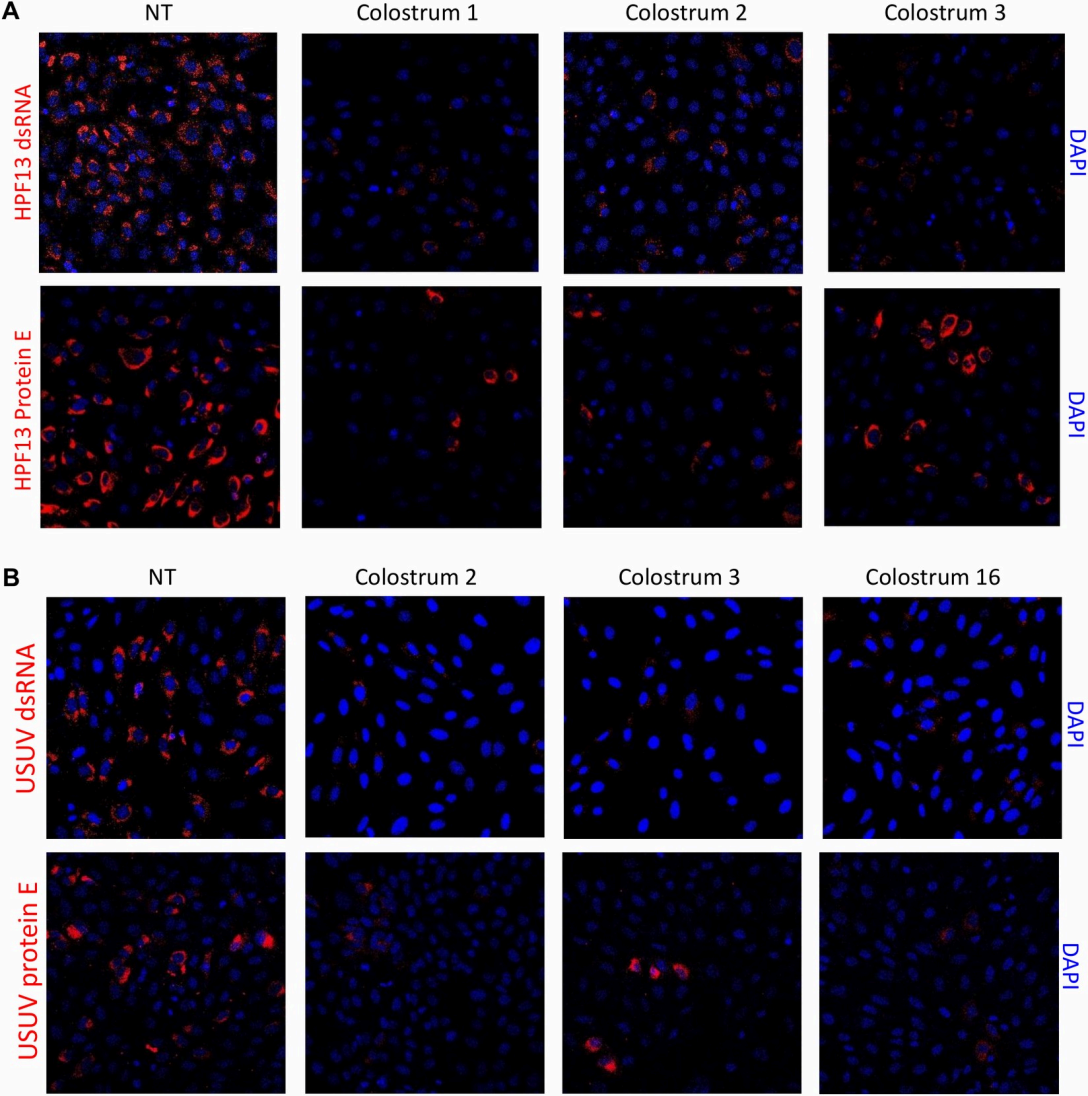
**Evaluation of the anti-ZIKV (A) and anti-USUV (B) activity of human colostrum with the immunofluorescence assay detecting the dsRNA intermediate of replication and the flavivirus protein E.** Cells and viruses (MOI = 3) were treated before and during the infection with the dilution of colostrum corresponding the ID_90_ in the virus inhibition assay. After 30 h of infection, cells were fixed and subjected to immunofluorescence.

To further confirm the antiviral action of human colostrum against ZIKV and USUV, immunofluorescence experiments detecting the dsRNA (an intermediate in flavivirus replication) and the flavivirus protein E were performed. As shown in Fig 2A and 2B, the synthesis of the dsRNA and the production of the protein E are significantly inhibited by colostrum for both viruses. The same results were obtained with the MR766 ZIKV strain (S3 Fig).

**Fig 3:**
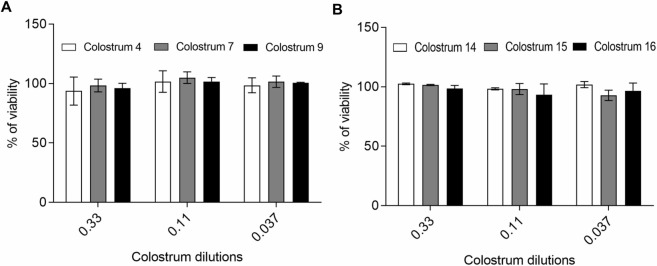
Evaluation of cell viability after colostrum treatment. Cells were treated under the same conditions of the ZIKV (A) and USUV (B) inhibition assay. Results obtained from 3 randomly selected colostrum samples are reported and indicated as % of untreated control. Values are means ± SEM of three independent experiments performed in duplicate.

Moreover, in order to exclude the possibility that the observed antiviral action was due to a cytotoxic effect, viability assays were performed by treating cells with human colostrum under the same experimental conditions of the virus inhibition assay described above. As expected, results indicated that colostrum aqueous fraction is not toxic for cells even at the lowest tested dilution (0.33). Results obtained with 3 randomly selected colostrum samples are reported in Fig 3.

Altogether, these results demonstrated that the aqueous fraction of human colostrum is intrinsically endowed with antiviral activity against two emerging flaviviruses without being toxic for cells *in vitro*.

Subsequently, we investigated the variations in the anti-ZIKV and anti-USUV activity of HM according to the different stages of lactation. To this aim, eleven mothers (Table 2) donated samples of colostrum, transitional and mature milk each.

**Table 2:** Main clinical characteristics of the second study group.

Sample n°	Gestational Age	Mother’s Age	Parity	Type of delivery
17	41+2	28	0000	CS
18	39+4	26	0000	S
19	39+6	33	1011	CS
20	39+4	30	0010	S
21	40+3	40	1001+1 VTP	S
22	37+1	33	1001	S
23	39+4	34	0000	S
24	38+5	40	1001	S
25	38+2	43	1001	CS
26	40+0	33	2002	S
27	40+4	41	0000	CS

S: spontaneous delivery; CS: cesarean section; VTP: voluntary termination of pregnancy

**Fig 4:**
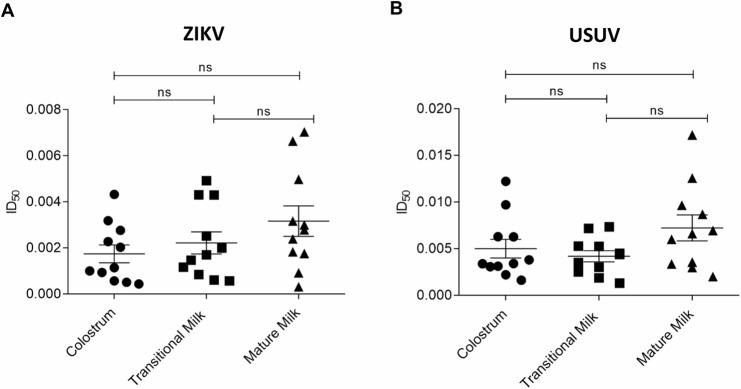
**Anti-ZIKV (A) and anti-USUV (B) activity of defatted human milk samples at different stages of maturation**. Cells and viruses were treated before and during the infection with serial dilutions of human milk aqueous fraction (from 1:3 to 1:6561 parts). The inhibitory dilution-50 values for colostrum, transitional milk and mature milk from a cohort of eleven mothers are reported. Results are expressed as mean ± SEM and analyzed by ANOVA followed by Bonferroni post hoc test.

First, the absence of cytotoxicity was verified for transitional and mature milk too (S4 Fig). The virus inhibition assays revealed that all samples exhibit net anti-ZIKV (Fig 4A) and anti-USUV activity (Fig 4B). Within each stage of lactation, milk samples exhibited a wide range of ID_50_s against both viruses, with mature milk showing the greatest variation. In the anti-ZIKV assays, the ID_50_ values ranged from 0.0004 to 0.004 in colostrum, from 0.0006 to 0.005 in transitional milk and from 0.0003 to 0.007 in mature milk (S2 Table). In the case of USUV, the ID_50_ values ranged from 0.002 to 0.01 in colostrum, from 0.001 to 0.007 in transitional milk and from 0.002 to 0.02 in mature milk (S3 Table). The mean antiviral activity of milk samples appeared to differ according to the stages of lactation with colostrum and transitional milk showing lower mean ID_50_ values than mature milk, but the difference did not reach statistical significance. These results also indicated that human milk is overall more active against ZIKV, confirming what previously demonstrated with colostrum samples and described above. In S5A and S5B Fig, the results obtained from every single mother are separately reported.

### Human colostrum alters the binding of ZIKV and USUV to cells

**Fig 5:**
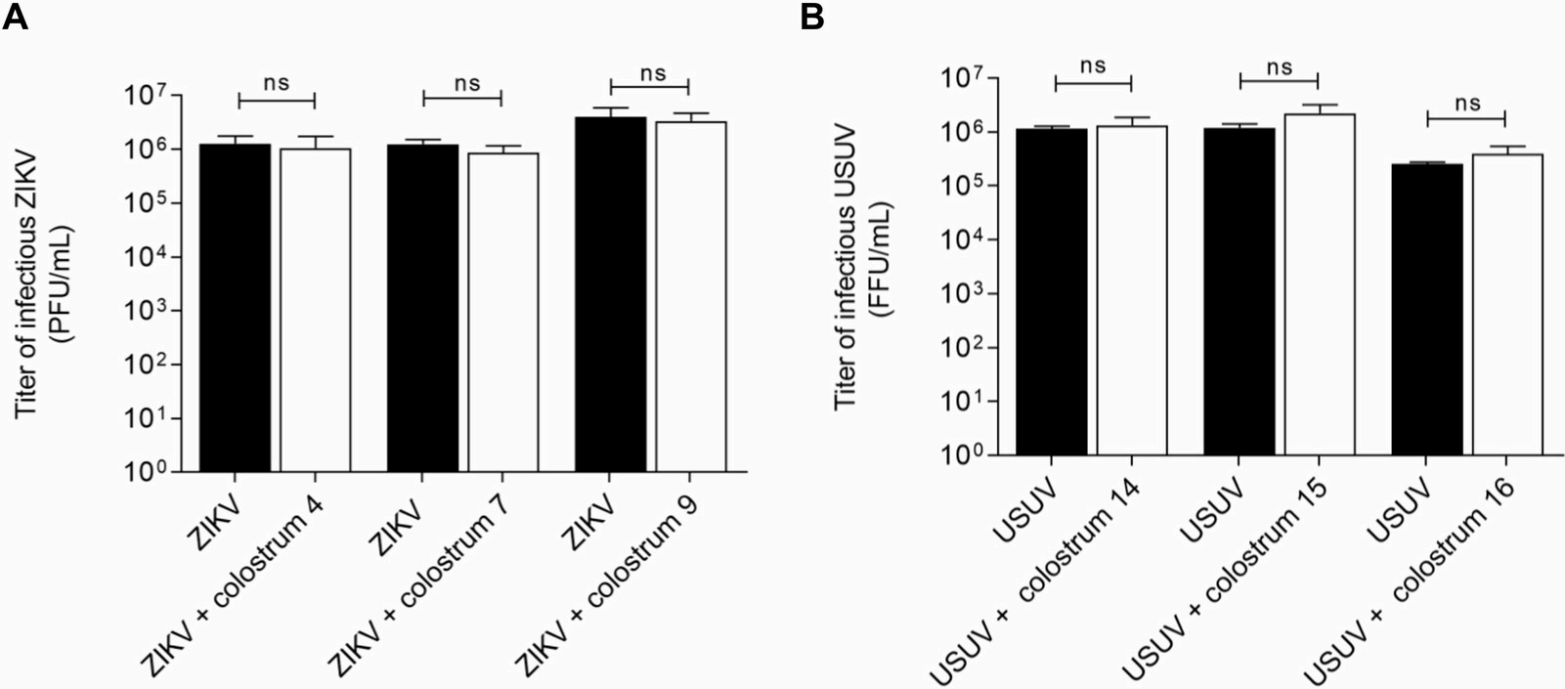
**Evaluation of ZIKV (A) and USUV (B) inactivation by human colostrum aqueous fraction.** Viruses were incubated with colostrum for 2h at 37°C and subsequently the residual viral infectivity was evaluated. On the y-axis, the infectious titers are expressed as plaque-forming units per ml (PFU/ml) (A) or focus-forming unit per ml (FFU/mL) (B). Error bars represent standard error of the mean of three independent experiments (Student’s t test; ns: not significant).

The second goal of this study is to investigate which step of ZIKV and USUV replicative cycle is inhibited by HM. Colostrum was selected for the execution of these tests due to the overall lower mean ID_50_ values compared to transitional and mature milk. Three samples of colostrum were randomly selected from the initial screening group for both viruses. First, we investigated whether the aqueous fraction of colostrum is endowed with an intrinsic virucidal activity, i.e. whether colostrum acts by directly inactivating the viral particle. As reported in Fig 5, we did not observe any significant virucidal activity for both viruses.

**Fig 6:**
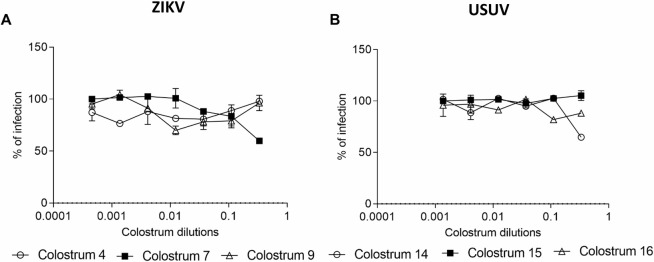
Pre-treatment assay. Cells were pre-treated with serial dilutions of colostrum for two hours before infection. After washing, cells were infected with ZIKV (A) or USUV (B) and the number of ZIKV plaques or USUV foci was evaluated after 72 or 24 hours respectively. The dose-response curves are reported. Data are presented as % of control. Values are means ± SEM of three independent experiments performed in duplicate.

The pre-treatment of cells with serial dilutions of colostrum for 2 h before infection did not alter ZIKV and USUV infectivity, thus indicating that colostrum does not act directly on cells preventing viral infection (Fig 6).

**Fig 7:**
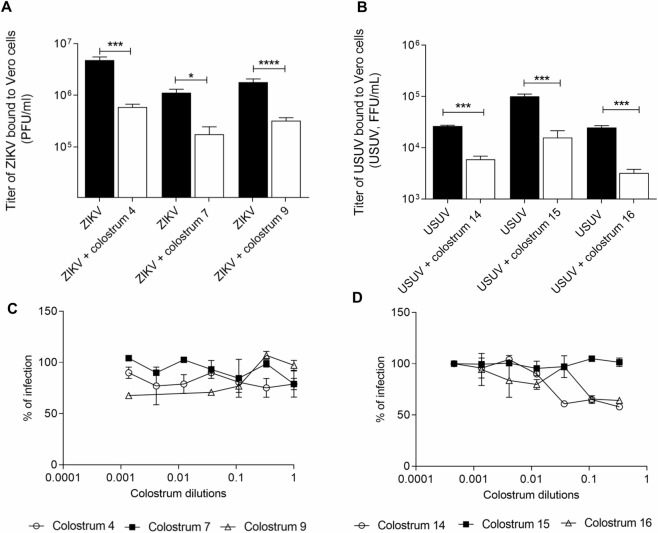
**Binding assay (A, B) and entry assay (C, D).** ZIKV (A) or USUV (B) (MOI = 3) were allowed to attach to the cells in presence of colostrum (ID_90_) for 2h on ice. The cell-bound virus titers were determined by means of plaque assay (ZIKV) or indirect immunostaining (USUV). On the y-axis, the infectious titers are expressed as plaque-forming units per ml (PFU/ml) (A) or focus-forming unit per ml (FFU/mL) (B). Error bars represent standard error of the mean of three independent experiments (Student’s t test; * p < 0.05; *** p < 0.001; ****p < 0.0001); For the entry assay, ZIKV (C) or USUV (D) were absorbed for 2 h at 4°C on pre-chilled Vero cells. After the removal of the unbound virus, the temperature was shifted to 37°C to allow the entry of pre-bound virus in presence of serial dilutions of colostrum for 2h. Unpenetrated virus was inactivated with citrate buffer and the number of ZIKV plaques or the number of USUV foci was evaluated after 72 h or 24 h respectively. The dose-response curves are reported. Data are presented as % of control. Values are means ± SEM of three independent experiments performed in duplicate.

Subsequently, the early steps of viral replication, i.e. the binding and the entry steps, were evaluated: the treatment with the aqueous fraction of colostrum was performed during the attachment of the virus to cells or during the cell-penetration processes. Results demonstrated that human colostrum does not alter the entry of viruses into cells (Fig 7C and 7D), but it acts by preventing the binding of both viruses to cells. In fact, as reported in Fig 7A and 7B, the titer of bound ZIKV and USUV to cells is significantly reduced in presence of colostrum. The reduction of ZIKV and USUV titer resulted in more than one order of magnitude between the treated and the untreated samples and was confirmed for all the tested colostrum samples (numerical results are reported in S4 Table).

**Fig 8:**
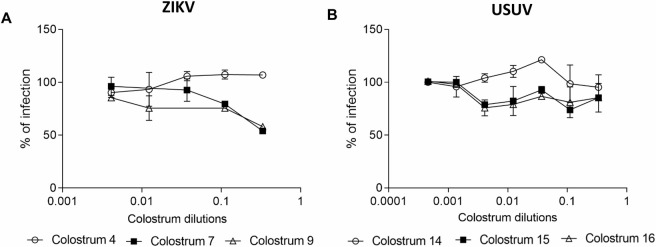
Post-entry assay. Cells were infected with ZIKV (A) or USUV (B) for 2 h at 37°C. After washing with citrate buffer, cells were treated with serial dilutions of colostrum for 3 h. The number of ZIKV plaques or USUV foci was evaluated after 72 or 24 hours respectively. The dose-response curves are reported. Data are presented as % of control. Values are means ± SEM of three independent experiments performed in duplicate.

Lastly, we verified the absence of any additional activity on the later steps of viral replication. When cells were treated with colostrum dilutions after ZIKV or USUV infection for three hours, no reduction in the number of infected cells was observed (Fig 8).

### Anti-ZIKV and anti-USUV components of HM: the HM-EVs and the HM-GAGs

Prompted by the above findings, we sought to identify new antiviral components of human milk that could contribute to the antiviral potency of this biofluid. To this aim EVs and GAGs were isolated and characterized.

Colostrum was selected as preferred lactation stage for EVs isolation, due to the previously reported higher concentration of EV in colostrum than transitional and mature milk [34].

**Fig 9:**
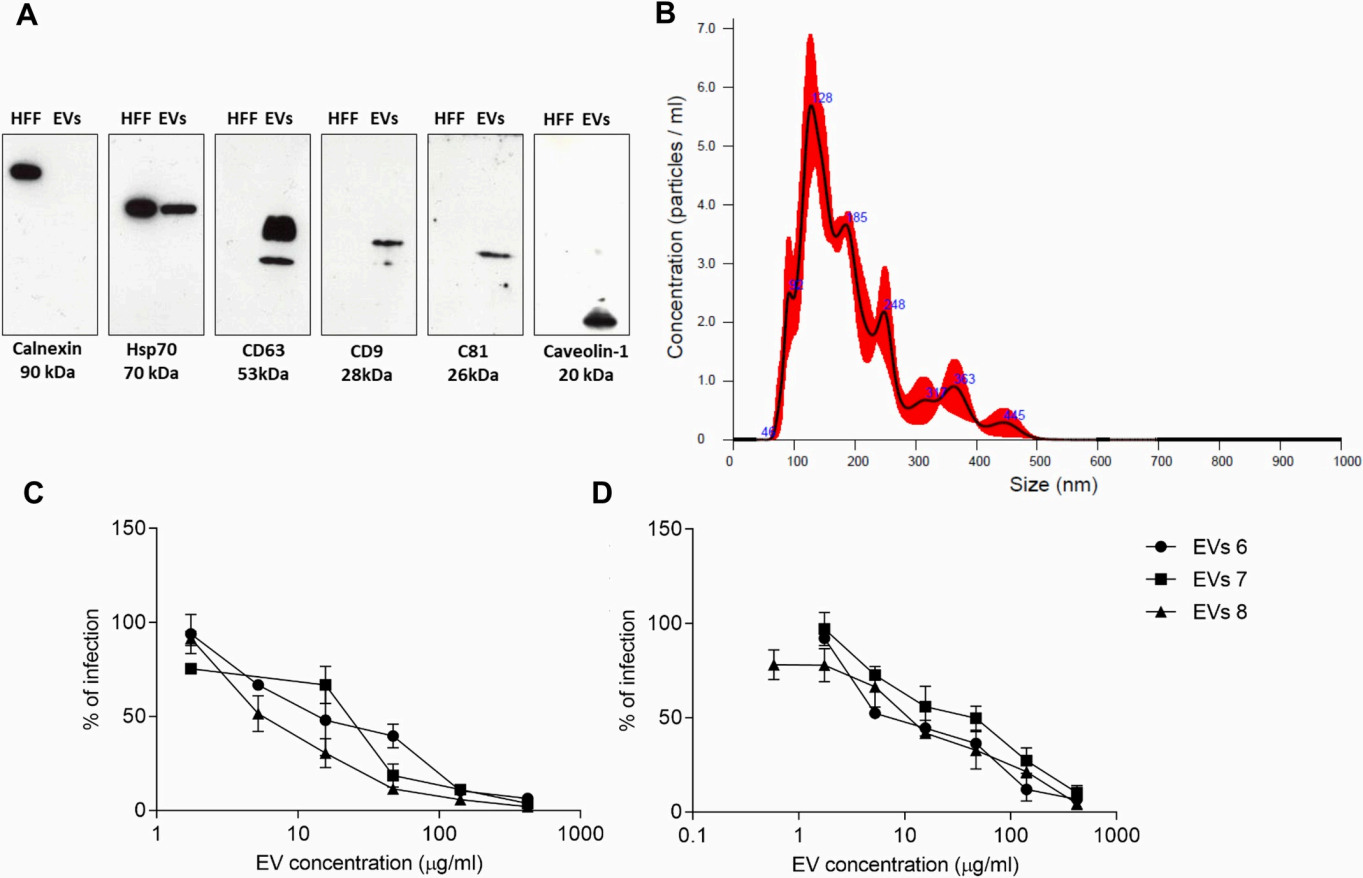
**Characterization of colostrum derived-EVs (A, B) and study of their anti-ZIKV (C) and anti-USUV (D) activity.** The protein profile of the colostrum-derived EVs was analysed by means of Western blotting using Abs against the endoplasmatic reticulum-related protein calnexin and against the EVs marker proteins CD63, CD9, CD81, Hsp70 and Caveolin-1. HFF cell lysate was used as control (A). In panel B, the Nanoparticle tracking analysis (NTA) is reported. In panels C and D the results obtained from the antiviral assays are reported. Cells and viruses were treated before and during the infection with serial dilutions of EVs. The dose response curves are reported. Data are presented as % of control. Values are means ± SEM of three independent experiments performed in duplicate.

The colostrum-derived EVs were characterized according to the “Minimal Information for Studies of Extracellular Vesicles guidelines” (MISEV2018) proposed by the International Society for Extracellular Vesicles (ISEV) [55]. As reported in Fig 9A, the EV-lysate was positive for the three tetraspanines CD63, CD9 and CD81, which are known to be enriched in EVs from multiple tissue sources, and it was positive for two cytosolic proteins (caveolin-1 and HSP70) usually recovered in EVs. Furthermore, the colostrum-derived EV lysate was negative for the contaminating endoplasmatic reticulum-related protein calnexin. We analyzed EV size and concentration by means of the nanoparticle tracking analysis. The NanoSight instrument showed that most EVs were between 100–400 nm in diameter: EVs had a mean diameter of 250.0 +/- 8.0 nm (EVs 6), 191.7 +/- 1.5 nm (EVs 7) and 200.3 +/- 2.9 nm (EVs 8) (Mode: 199.4 +/- 16.0 nm, 130.5 +/- 7.9 nm, 137.5 +/- 14.6 nm for sample 6, 7 and 8 respectively). Particle concentration was 7.55 x10^12^ (EVs 6), 7.02 x10^12^ (EVs 7) and 9.33 x 10^12^ (EVs 8) particles/ml. A representative analysis is reported in Fig 9B (S6 Fig reports the NTA analysis of EVs 6 and EVs 8).

After the characterization, EVs were tested *in vitro* against ZIKV and USUV. The results (Fig 9C and 9D) demonstrated that colostrum-derived EVs are endowed with a strong antiviral activity against both viruses. Notably, the EC_50_ values of the 3 EV populations were 11.47, 7.04 and 16.22 μg protein/ml for ZIKV and 11.61, 12.33 and 32.57 μg protein/ml for USUV.

**Fig 10:**
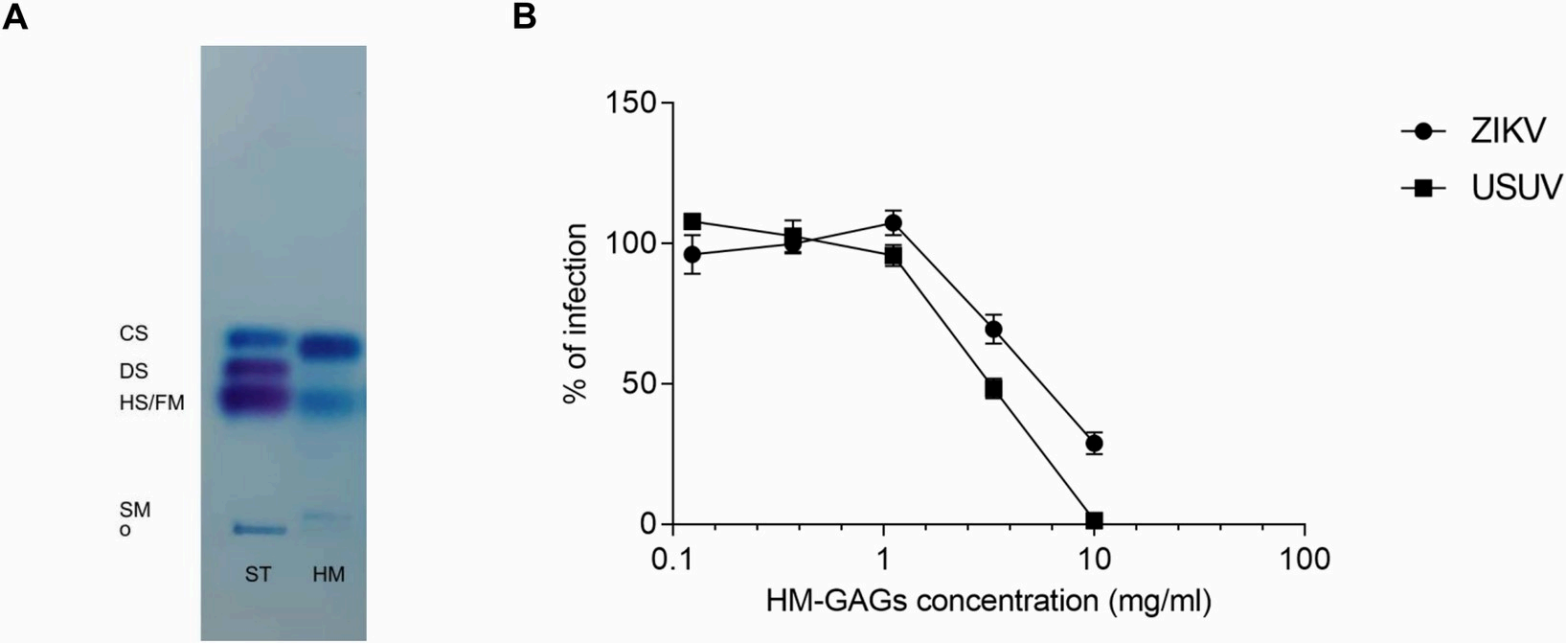
**Electrophoresis separation of HM-GAGs (A) and evaluation of their anti-ZIKV and anti-USUV action (B).** (A) Purified HM-GAGs were separated by means of acetate of cellulose electrophoresis. CS: chondroitin sulfate; DS: dermatan sulfate; HS: heparan sulfate; FM: fast-moving heparin; SM: slow-moving heparin; o: origin; ST: GAGs standard; HM: human milk; (B) Cells and viruses were treated before and during the infection with serial dilutions of HM-GAGs. Data are presented as % of control and the dose response curves are reported. Values are means ± SEM of three independent experiments performed in duplicate.

Next, the antiviral potency of GAGs isolated from a pool of mature milk samples was evaluated *in vitro*. Mature milk was selected as preferred biological matrix because is more commonly available than colostrum. According to previous studies [39,40,56], the HM-GAGs fraction tested in this study was mainly composed of CS/DS and HS/Hep as evident from the electrophoresis (Fig 10A). Moreover, from the structural characterization analysis (confirmed by the electrophoresis), we obtained a percentage of ~55% CS and 1–2% DS, ~40% HS/low-sulfated Hep (known as fast-moving Hep) and ~2% high-sulfated Hep (known as slow-moving Hep), and trace amount (1–2%) of HA. Additionally, the HM-CS was confirmed to have a very typical low charge density (~0.35) compared to the other known CS [57]. Finally, the purified HM-GAGs were tested to have a purity greater than 98%.

Results from the antiviral assays revealed that HM-GAGs are active against ZIKV and USUV in the range of physiological concentrations detected in term and preterm human colostrum [42] (Fig 10B). The EC_50_ values were 5.8 mg/ml for ZIKV and 3.3 mg/ml for USUV with the higher concentration tested (10 mg/ml) able to completely inhibit USUV infection and to inhibit the 70% of ZIKV infection.

Both EVs samples and HM-GAGs did not show any cytotoxicity (S7 Fig).

Altogether these results demonstrated that colostrum derived EVs and HM-GAGs contribute, at least in part, to the anti-ZIKV and anti-USUV intrinsic action of human milk.

## Discussion and conclusion

While the antiviral activity of HM and its components against numerous viral pathogens have been described in literature, its role in protecting against emerging arboviruses has been poorly investigated so far [25,58,59]. In this study we addressed this issue focusing on two emerging flaviviruses: we assessed the anti-ZIKV and the anti-USUV activity of human milk in its different stages of maturation and we explored the antiviral contribution of specific components, namely the HM-EVs and the HM-GAGs.

The first notable finding is that HM is endowed with antiviral activity against ZIKV and USUV in all the stages of lactation (colostrum, transitional and mature milk) with no significant differences between them. We previously reported a different pattern in the anti-CMV activity of human milk [54]: we demonstrated that colostrum from CMV-IgG+ mothers was significantly more potent than transitional and mature milk and this was due to the higher content of specific immune factors, especially sIgA, in the early stages of lactation. On the contrary, the presence of specific immunoglobulins in the milk samples of the present study is very unlikely, considering the absence of a past travel history in ZIKV endemic areas of the donor mothers and the low seroprevalence of USUV in Europe [11]. The anti-ZIKV and anti-USUV action is attributable to non-specific bioactive factors that act independently from the mother serostatus. Which is the antiviral potency of colostrum from ZIKV or USUV infected mothers remains an interesting open question.

We also demonstrated that human colostrum exerts an antiviral action against two different ZIKV strains, the HPF2013 and the MR766, belonging to the Asian and African lineage respectively. These results indicate that breast milk could play a protective role against either the microcephalic Asian strains, that have caused the latest epidemics, and the African strains, that have shown to be more infectious *in vitro* and *in vivo*, but have not caused any recently reported human case [60]. To the best of our knowledge, this is the first study reporting the intrinsic anti-USUV activity of human milk. On the contrary, Pang et al. recently indicated a potential anti-ZIKV action of HM in an artificial feeding model mouse [25] and other two reports demonstrated that breast milk is able to inactivate ZIKV in a time-dependent manner. These latter studies attributed the antiviral potential to the fat containing cream fraction of human milk, in which the free fatty acids, released upon storage by milk lipases in a time-dependent manner, incorporate into the viral envelope thereby destroying the viral particle [30,61]. In our experiments, we eliminated the storage affected lipid components from the milk samples and we analyzed the aqueous fractions after having verified that our storage method did not alter the antiviral properties. Our results therefore indicate the presence of an intrinsic anti-ZIKV activity in the aqueous fraction of HM too.

We explored the anti-ZIKV and anti-USUV activity of human milk analyzing the putative step of viral replication inhibited by this biofluid. We focused on the first stage of lactation, because colostrum samples collectively showed the lowest ID_50_ values in the virus inhibition assays. We demonstrated that the aqueous fraction of colostrum acts by altering the binding of ZIKV and USUV to cells, thus preventing cellular infection. Despite the early steps of USUV and ZIKV replicative cycles are not yet fully defined, it has been demonstrated that different molecules play a role in flaviviruses attachment to cells. The most common attachment factors are negatively charged glycosaminoglycans (GAGs), which can be utilized by several flaviviruses, including DENV, WNV, JEV and tick-borne encephalitis virus (TBEV), as low-affinity attachment factors to concentrate the virus on cell surface [62]. Contradictory results have emerged instead from studies about the dependence of ZIKV on cellular GAGs [63–66]. In addition, phosphatidylserine (PS) receptors’ families, such as T-cell immunoglobulin (TIM) and TYRO3, AXL and MERTK (TAM), as well as integrins and C-type lectin receptors (CLRs), have been described as key factors during the initial steps of flavivirus cell invasion [62,67]. Given the extremely complex composition of human milk and the results obtained from the study of its mechanism of action, we could hypothesize the presence of one or more factors in HM able to limit USUV and ZIKV interaction with the above-mentioned cellular receptors. We can exclude a possible action of residual free fatty acids, since the mechanism of action indicated by Conzelmann et al. and attributed to the lipid components consisted of a virucidal activity.

To support our results, we investigated the antiviral activity of two HM components derived from the aqueous fraction of human milk. HM-EVs showed a strong inhibition of ZIKV and USUV infection *in vitro*, suggesting that they could contribute, at least in part, to the overall antiviral action of human milk. Consistently with these findings, previous studies demonstrated the antiviral potency of HM-EVs and indicated that they compete with viruses for the binding to cellular receptors [44,68]. In particular, Näslund and colleagues showed that HM-exosomes compete with HIV-1 for binding to DC-SIGN receptor on monocyte-derived dendritic cells. Even though our results are preliminary and the mechanism of action has not been investigated yet, we speculate that a similar mechanism could be possible against ZIKV and USUV too.

GAGs are other HM components that could contribute in inhibiting the binding of both flaviviruses to cells. Since numerous viruses, including some flaviviruses, attach to cell exploiting cellular GAGs, these complex carbohydrates from different origins have been largely investigated for their ability to act as soluble receptors [69–74]. Notably, the first data available on the antiviral role of GAGs isolated from HM are those reported by Newburg et al. demonstrating that HM-CS was able to inhibit the binding of the HIV envelop glycoprotein gp120 to the cellular CD4 receptor [46]. In this study, HM-GAGs were tested against ZIKV and USUV at concentrations that would be relevant to breastfed infants [40]. In particular, highest GAGs values are present at 4^th^ day after partum (~ 9.3 mg/ml and ~ 3.8 mg/ml in preterm and term milk, respectively), followed by a progressive decrease up to day 30^th^ (~ 4.3 mg/ml and ~ 0.4 mg/ml). Given the possibility that cellular GAGs are low-affinity attachment factors for ZIKV and USUV, the antiviral action of HM-GAGs (potentially acting as decoy receptors) is not as strong as expected, but the EC_50_ values fall within the range of GAG concentrations in preterm and term colostrum. Furthermore, the GAGs concentration detected in preterm mature milk is still partially active against USUV. These results indicate that HM-GAGs could only partially contribute to the overall antiviral action of human milk, mostly acting during the first days after birth. This also highlights that the dependence of ZIKV and USUV on cellular GAGs for their attachment to cells warrant further investigations.

In conclusion, the intrinsic anti-ZIKV activity of human milk here reported, along with the higher viscosity detected in colostrum from ZIKV-infected mothers and the storage-dependent virucidal activity of HM lipid components, are three different factors that hinder the spread of ZIKV through breastfeeding [30,75]. Furthermore, we can now add USUV to the list of viral pathogens inhibited by human milk. Altogether, our data support the WHO recommendations about breast-feeding during ZIKV infection and could contribute to producing new guidelines for a possible USUV epidemic.

## Supporting information

S1 Table Anti-ZIKV and anti-USUV activities of defatted colostrum (numerical results of Fig 1A). (DOCX)

S2 Table ID_50_ values of defatted human milk samples at different stages of maturation against ZIKV (numerical results of Fig 5A). (DOCX)

S3 Table ID_50_ values of defatted human milk samples at different stages of maturation against USUV (numerical results of Fig 5B). (DOCX)

S4 Table Numerical results of the binding assays reported in Fig 8A and 8B (the mean values are reported). (DOCX)

S1 Fig **Anti-ZIKV (A) and anti-USUV (B) activities of two fresh colostrum samples.** Cells and viruses were treated before and during the infection with serial dilutions of colostrum aqueous fraction (from 1:3 to 1:6561 parts). The dose-response curves are reported. Data are presented as % of control. Values are means ± SEM of three independent experiments performed in duplicate. The ID_50_ values obtained from the ZIKV antiviral assays values were 0.0037 (colostrum F1) and 0.00097 (colostrum F2). In the case of USUV, the ID_50_ values were 0.018 (colostrum F1) and 0.02 (colostrum F2).
(PDF)

S2 Fig Comparison between the antiviral activity of colostrum from term and preterm mothers. Cells and viruses were treated before and during the infection with serial dilutions of human colostrum aqueous fraction (from 1:3 to 1:6561 parts). Anti-ZIKV and anti-USUV inhibitory dilution-50 values obtained from three independent experiments are reported and stratified to compare term and preterm mothers. Panel A reports the results obtained by testing colostra against ZIKV, while panel B shows the results for USUV. Results are expressed as mean ± SEM of inhibitory dilution-50 values (Student’s t test; ns: not significant).
(PDF)

S3 Fig Evaluation of the anti-ZIKV activity of human colostrum against the MR766 strain with immunofluorescence assays detecting the dsRNA and the flavivirus protein E. Cells and viruses (MOI = 3) were treated before and during the infection with the dilution of colostrum corresponding the ID_90_ in the virus inhibition assay. After 30 h of infection, cells were fixed and subjected to immunofluorescence.
(PDF)

S4 Fig **Evaluation of cell viability after the treatment with transitional (A, C) or mature milk (B, D).** Cells were treated under the same conditions of the ZIKV (A, B) and USUV (C, D) inhibition assays. Results from 3 randomly selected samples are reported in each graph. Data are indicated as % of untreated control. Values are means ± SEM of three independent experiments performed in duplicate.
(PDF)

S5 Fig **Anti-ZIKV (A) and anti-USUV (B) activity of defatted human milk samples at different stages of maturation**. The inhibitory dilution-50 values of colostrum, transitional and mature milk obtained from every single mother are separately reported indicating the sample number.
(PDF)

S6 Fig Nanoparticle tracking analysis (NTA) of EVs 6 (A) and EVs 8 (B). (PDF)

S7 Fig **Evaluation of cell viability after EVs (A) or GAGs treatment (B).** Cells were treated under the same experimental conditions of the ZIKV and USUV inhibition assay, but without infection. Cell viability was evaluated after 24 h or 72 h, respecting the same experimental timing of USUV or ZIKV antiviral assay respectively. Results obtained with one representative EV population and with the GAGs preparation are reported and indicated as % of untreated control. Values are means ± SEM of three independent experiments performed in duplicate.
(PDF)
