# Supplementary material for: Anti-Zika virus and anti-Usutu virus activity of human milk and its components
Source: PLoS Negl Trop Dis. 2020 Oct 7;14(10):e0008713. doi: 10.1371/journal.pntd.0008713 (PMC7571670; doi:10.1371/journal.pntd.0008713)
Supplement: S1 Table — (DOCX) [file pntd.0008713.s001.docx]

**S1 Table. Anti-ZIKV and anti-USUV activities of defatted colostrum (numerical results of**

**Fig 1A)**

| **Sample n°** | **ID_50_ against ZIKV**  **(best-fit value)** | **ID_50_ against USUV (best-fit value)** |
| --- | --- | --- |
| 1 | 0,0006686 | / |
| 2 | 0,000454 | 0,007356 |
| 3 | 0,001033 | 0,003179 |
| 4 | 0,001083 | 0,003747 |
| 5 | 0,002688 | / |
| 6 | 0,0009027 | 0,006078 |
| 7 | 0,001676 | 0,01172 |
| 8 | 0,002508 | 0,007228 |
| 9 | 0,0002905 | / |
| 10 | 0,0004075 | 0,002194 |
| 11 | 0,001844 | / |
| 12 | / | 0,03555 |
| 13 | / | 0,004581 |
| 14 | / | 0,008625 |
| 15 | / | 0,008683 |
| 16 | / | 0,00417 |
| F1 | 0,0037 | 0,01831 |
| F2 | 0,0009657 | 0,02006 |

ID_50_: inhibitive dilution producing a 50% reduction of infection

F1, F2: Fresh colostrum samples

/: not tested
