# Supplementary material for: Anti-Zika virus and anti-Usutu virus activity of human milk and its components
Source: PLoS Negl Trop Dis. 2020 Oct 7;14(10):e0008713. doi: 10.1371/journal.pntd.0008713 (PMC7571670; doi:10.1371/journal.pntd.0008713)
Supplement: S2 Table — (DOCX) [file pntd.0008713.s002.docx]

**S2 Table. ID_50_ values of defatted human milk samples at different stages of maturation against ZIKV (numerical results of Fig 5A)**

|  | **ID_50_ values against ZIKV**  **(best-fit value)** | | | **F-test** | | |
| --- | --- | --- | --- | --- | --- | --- |
| **Sample n°** | **COL** | **TM** | **MM** | **COL**  **vs**  **TM** | **COL**  **vs**  **MM** | **TM**  **vs**  **MM** |
| 17 | 0.0005032 | 0.001461 | 0.003156 | *** | *** | *** |
| 18 | 0.0005609 | 0.001156 | 0.004969 | ** | *** | *** |
| 19 | 0.002273 | 0.004295 | 0.002382 | * | ns | * |
| 20 | 0.003174 | 0.004287 | 0.007032 | ns | ** | ** |
| 21 | 0.001135 | 0.001693 | 0.002777 | ns | *** | * |
| 22 | 0.001135 | 0.001693 | 0.002777 | *** | ** | ns |
| 23 | 0.000427 | 0.0008354 | 0.001824 | *** | *** | *** |
| 24 | 0.002755 | 0.00199 | 0.006628 | ns | ** | ** |
| 25 | 0.0009303 | 0.002509 | 0.00299 | *** | *** | ns |
| 26 | 0.0009941 | 0.000566 | 0.0003018 | ns | ** | ns |
| 27 | 0.004317 | 0.004913 | 0.001753 | ns | ** | ** |

ID_50_: inhibitive dilution producing a 50% reduction of infection; COL: colostrum; TM: transitional milk; MM: mature milk; F-test: Fisher test; *** p<0.001;

** p<0.01; * p<0.05; ns: not significant
