# Supplementary material for: Anti-Zika virus and anti-Usutu virus activity of human milk and its components
Source: PLoS Negl Trop Dis. 2020 Oct 7;14(10):e0008713. doi: 10.1371/journal.pntd.0008713 (PMC7571670; doi:10.1371/journal.pntd.0008713)
Supplement: S3 Table — (DOCX) [file pntd.0008713.s003.docx]

**S3 Table. ID_50_ values of defatted human milk samples at different stages of maturation against USUV (numerical results of Fig 5B)**

|  | **ID_50_ values against USUV**  **(best-fit value)** | | | **F-test** | | |
| --- | --- | --- | --- | --- | --- | --- |
| **Sample n°** | **COL** | **TM** | **MM** | **COL**  **vs**  **TM** | **COL**  **vs**  **MM** | **TM**  **vs**  **MM** |
| 17 | 0,002186 | 0,003496 | 0,01255 | ns | *** | *** |
| 18 | 0,006276 | 0,004361 | 0,006926 | ns | ns | ns |
| 19 | 0,003785 | 0,007153 | 0,001984 | * | * | ** |
| 20 | 0,006256 | 0,004495 | 0,01718 | ns | *** | *** |
| 21 | 0,003375 | 0,005256 | 0,009635 | ns | ** | * |
| 22 | 0,0122 | 0,001291 | 0,002958 | *** | *** | ** |
| 23 | 0,003051 | 0,002494 | 0,006594 | ns | ** | *** |
| 24 | 0,0034 | 0,001854 | 0,008679 | * | *** | *** |
| 25 | 0,003111 | 0,005222 | 0,003545 | ** | ns | * |
| 26 | 0,001616 | 0,002995 | 0,003334 | ** | ** | ns |
| 27 | 0,009698 | 0,007331 | 0,005985 | ns | ns | ns |

ID_50_: inhibitive dilution producing a 50% reduction of infection; COL: colostrum; TM: transitional milk; MM: mature milk; F-test: Fisher test; *** p<0.001;

** p<0.01; * p<0.05; ns: not significant
