# Supplementary material for: Anti-Zika virus and anti-Usutu virus activity of human milk and its components
Source: PLoS Negl Trop Dis. 2020 Oct 7;14(10):e0008713. doi: 10.1371/journal.pntd.0008713 (PMC7571670; doi:10.1371/journal.pntd.0008713)
Supplement: S4 Table — (DOCX) [file pntd.0008713.s004.docx]

**S4 Table. Numerical results of the binding assays reported in Fig 8A and 8B (the mean values are reported)**

| **ZIKV** | | | |
| --- | --- | --- | --- |
|  | **Colostrum 4** | **Colostrum 7** | **Colostrum 9** |
| **Untreated** | 4797000 PFU/ml | 1121000 PFU/ml | 1779643 PFU/ml |
| **Treated** | 590250 PFU/ml | 176500 PFU/ml | 323200 PFU/ml |

| **USUV** | | | |
| --- | --- | --- | --- |
|  | **Colostrum 14** | **Colostrum 15** | **Colostrum 16** |
| **Untreated** | 26370 FFU/ml | 100541 FFU/ml | 24745 FFU/ml |
| **Treated** | 5897 FFU/ml | 15701 FFU/ml | 3199 FFU/ml |
