# Supplementary figures and images for: Anti-Zika virus and anti-Usutu virus activity of human milk and its components
Source: PLoS Negl Trop Dis. 2020 Oct 7;14(10):e0008713. doi: 10.1371/journal.pntd.0008713 (PMC7571670; doi:10.1371/journal.pntd.0008713)

**A**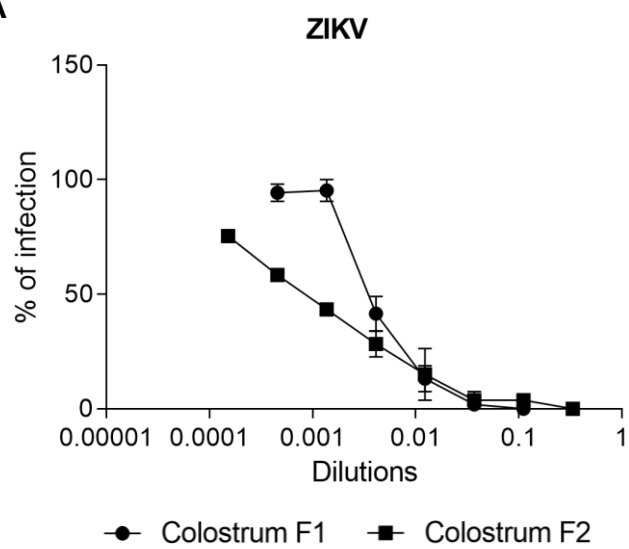**B**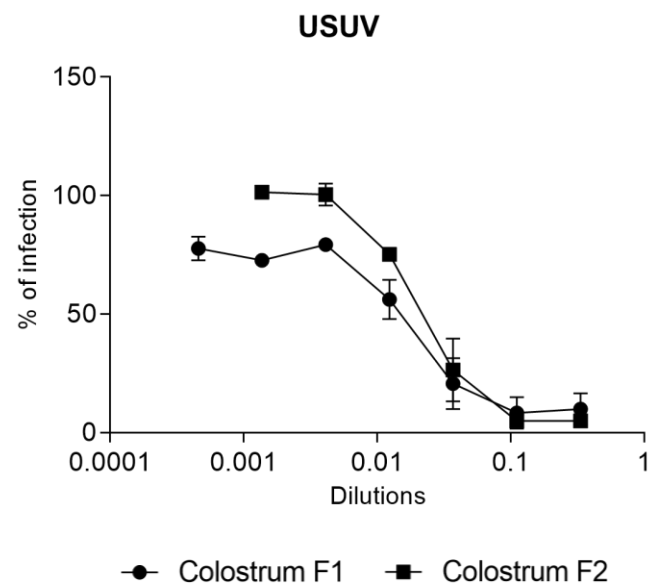

Supplement: S1 Fig — Anti-ZIKV (A) and anti-USUV (B) activities of two fresh colostrum samples. Cells and viruses were treated before and during the infection with serial dilutions of colostrum aqueous fraction (from 1:3 to 1:6561 parts). The dose-response curves are reported. Data are presented as % of control. Values are means ± SEM of three independent experiments performed in duplicate. The ID50 values obtained from the ZIKV antiviral assays values were 0.0037 (colostrum F1) and 0.00097 (colostrum F2). In the case of USUV, the ID50 values were 0.018 (colostrum F1) and 0.02 (colostrum F2). (PDF) [file pntd.0008713.s005.pdf]

**A**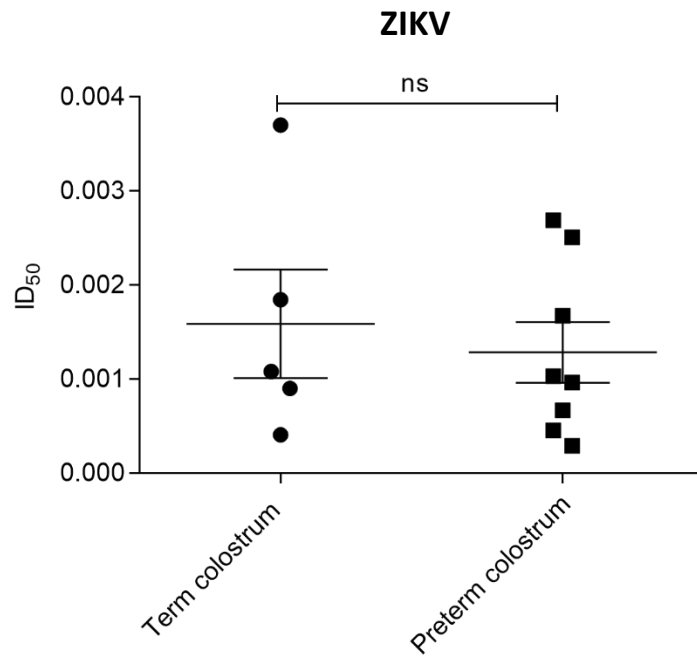**B**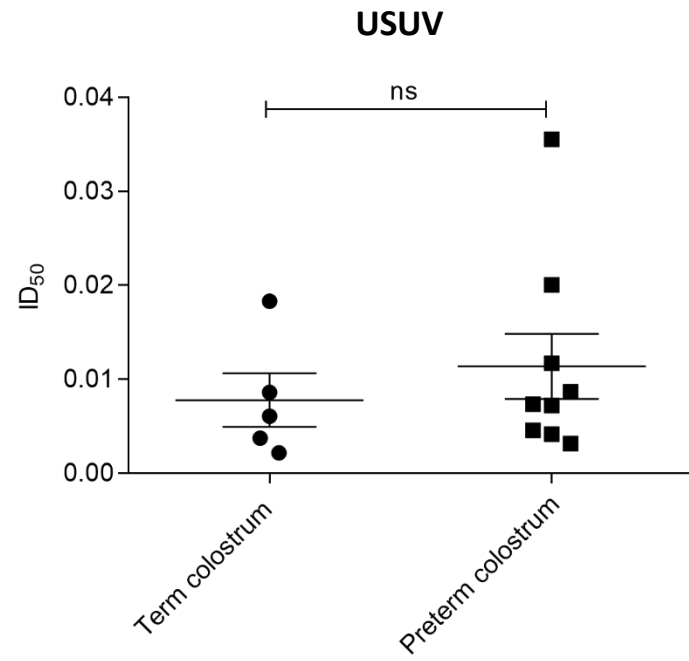

Supplement: S2 Fig — Cells and viruses were treated before and during the infection with serial dilutions of human colostrum aqueous fraction (from 1:3 to 1:6561 parts). Anti-ZIKV and anti-USUV inhibitory dilution-50 values obtained from three independent experiments are reported and stratified to compare term and preterm mothers. Panel A reports the results obtained by testing colostra against ZIKV, while panel B shows the results for USUV. Results are expressed as mean ± SEM of inhibitory dilution-50 values (Student’s t test; ns: not significant). (PDF) [file pntd.0008713.s006.pdf]

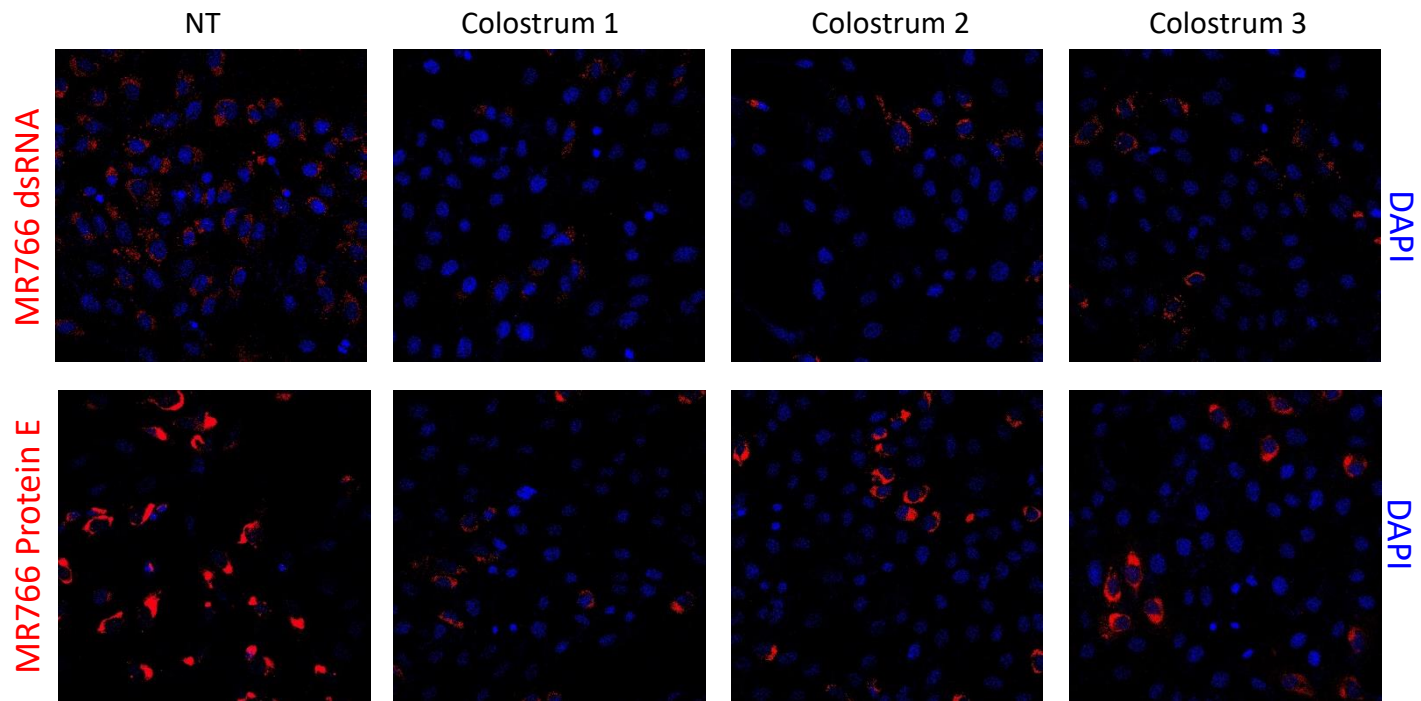

Supplement: S3 Fig — Cells and viruses (MOI = 3) were treated before and during the infection with the dilution of colostrum corresponding the ID90 in the virus inhibition assay. After 30 h of infection, cells were fixed and subjected to immunofluorescence. (PDF) [file pntd.0008713.s007.pdf]

**A**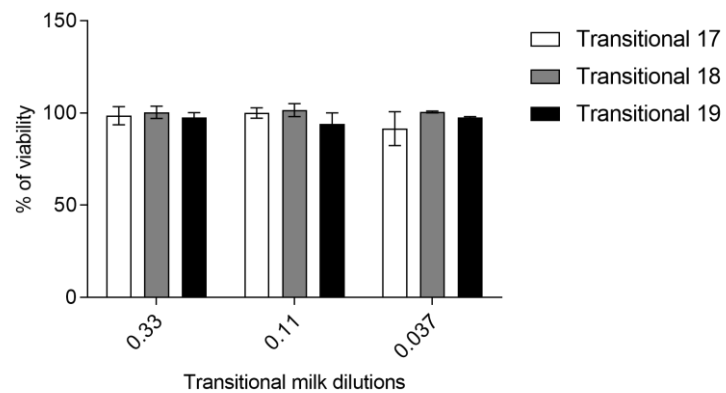**B**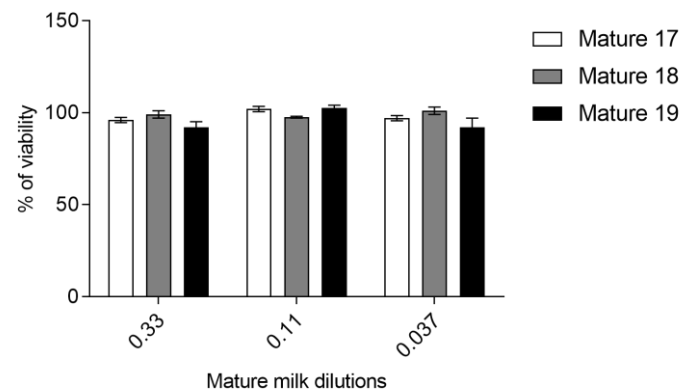**C**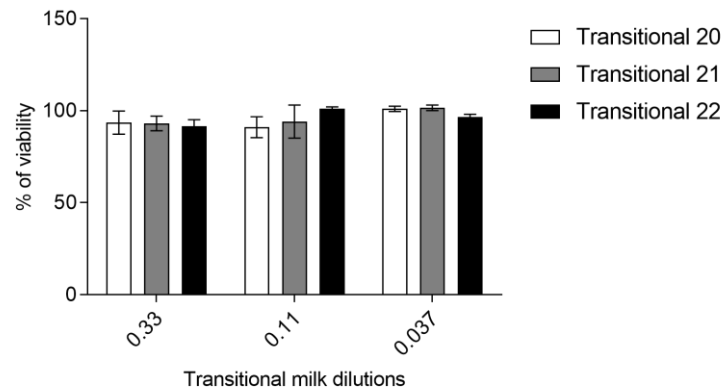**D**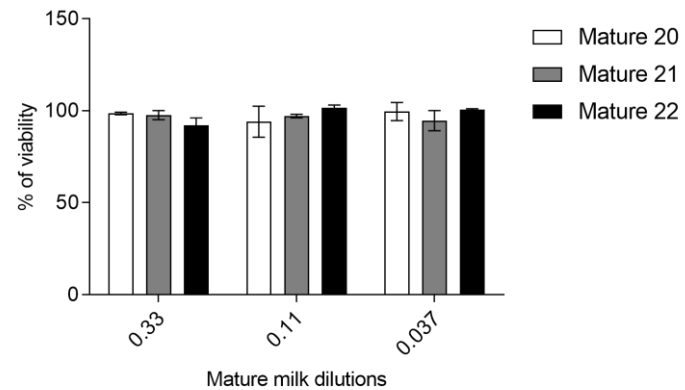

Supplement: S4 Fig — Evaluation of cell viability after the treatment with transitional (A, C) or mature milk (B, D). Cells were treated under the same conditions of the ZIKV (A, B) and USUV (C, D) inhibition assays. Results from 3 randomly selected samples are reported in each graph. Data are indicated as % of untreated control. Values are means ± SEM of three independent experiments performed in duplicate. (PDF) [file pntd.0008713.s008.pdf]

**A**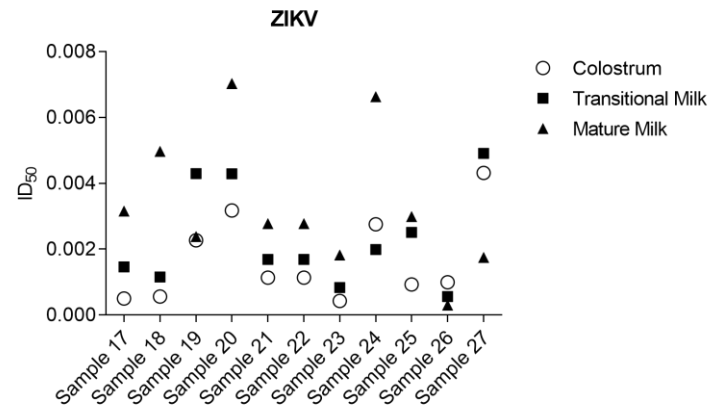**B**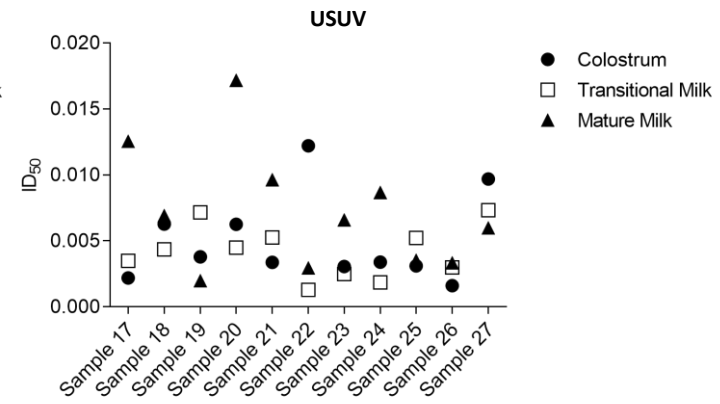

Supplement: S5 Fig — Anti-ZIKV (A) and anti-USUV (B) activity of defatted human milk samples at different stages of maturation. The inhibitory dilution-50 values of colostrum, transitional and mature milk obtained from every single mother are separately reported indicating the sample number. (PDF) [file pntd.0008713.s009.pdf]

**A**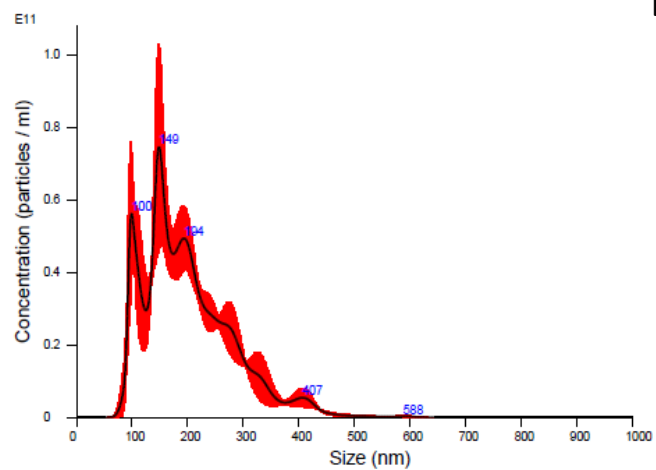**B**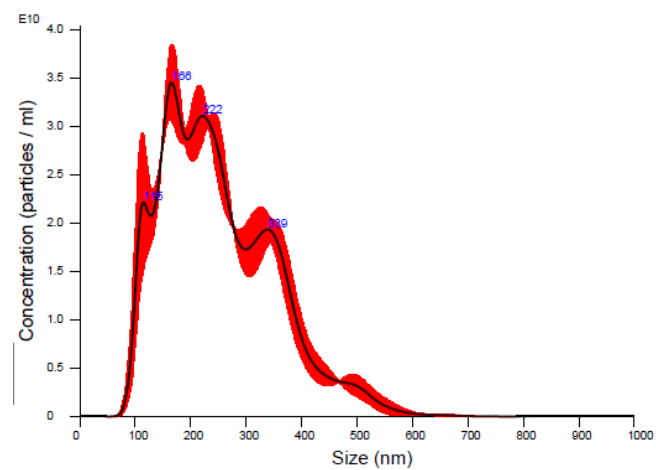

Supplement: S6 Fig — (PDF) [file pntd.0008713.s010.pdf]

**A**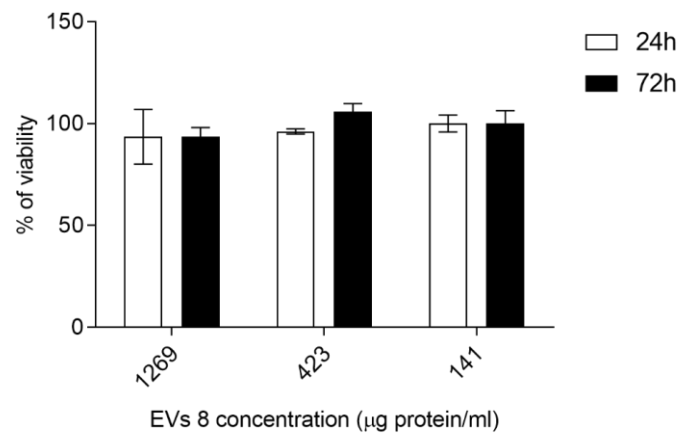**B**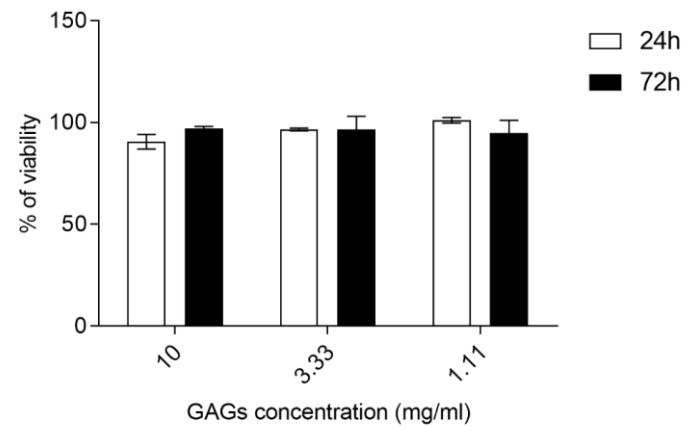

Supplement: S7 Fig — Evaluation of cell viability after EVs (A) or GAGs treatment (B). Cells were treated under the same experimental conditions of the ZIKV and USUV inhibition assay, but without infection. Cell viability was evaluated after 24 h or 72 h, respecting the same experimental timing of USUV or ZIKV antiviral assay respectively. Results obtained with one representative EV population and with the GAGs preparation are reported and indicated as % of untreated control. Values are means ± SEM of three independent experiments performed in duplicate. (PDF) [file pntd.0008713.s011.pdf]
